# EQ-5D-5L measurement properties are superior to EQ-5D-3L across the continuum of health using US value sets

**DOI:** 10.1186/s12955-022-02031-8

**Published:** 2022-09-09

**Authors:** Ruixuan Jiang, Kim Rand, Maja Kuharic, A. Simon Pickard

**Affiliations:** 1 Center for Observational and Real-World Evidence, Merck & Co., Rahway, NJ 07065 USA; 2 The Health Services Research Unit - HØKH, Akershus University Hospital, Lørenskog, Norway; 3 Maths in Health, Rotterdam, The Netherlands; 4 Department of Pharmacy Systems, Outcomes, and Policy, College of Pharmacy, University of Illinois at Chicago, 833 South Wood St, MC 871, Chicago, IL 60612 USA

**Keywords:** EQ-5D, Value set, Health technology assessment, Health measurement

## Abstract

**Objective:**

The objective of this study was to compare the measurement properties of the US EQ-5D-3L, EQ-5D-5L, and -5L to -3L crosswalk value sets (3L; 5L; 5L > 3L) across the spectrum of health.

**Methods:**

The three scoring approaches were compared in terms of range of scale, percent of worse-than-dead health states, and mean single-level transitions. Discriminative ability was compared by leveraging two cross-sectional datasets. A novel method was used to visualize and compare the responsiveness of 3L and 5L scoring approaches across EQ VAS values.

**Results:**

The US 5L value set had the broadest range of scale at 1.573 (vs. 1.109 for 3L and crosswalk). The crosswalk had the smallest mean single-level transition of 0.061 (vs. 0.078 for 5L and 0.111 for 3L). The 5L value set tended to be more discriminative/greater statistical efficiency than the crosswalk (F-statistic ratio: 1.111, 95% CI 0.989–1.240) and 3L (F-statistic ratio: 1.102 95% CI 0.861–1.383) across levels of general health. The 5L was the most responsive value set between EQ VAS values of 25 and 75.

**Conclusion:**

These results imply greater sensitivity of the 5L to health changes and potentially lower incremental cost-utility ratios compared to the 3L.

**Supplementary Information:**

The online version contains supplementary material available at 10.1186/s12955-022-02031-8.

## Highlights

- This study is among the first to compare the performance of value sets for the US to help users understand health utility gains depending on the value set/scoring approach selected.
- The 5L value set had improved interval-level measurement properties than the 3L and 3L > 5L value sets.
- Empirical analyses, including a novel simulation method, showed that the 5L value set tended to have greater discriminative ability across the entire health continuum.

**Supplementary Information:**

The online version contains supplementary material available at 10.1186/s12955-022-02031-8.

## Background

Health technology assessment (HTA) is predicated upon methodologies and decision-making criteria that inform reimbursement and assess the value of competing health care technologies. In addition to survival benefits, it is essential to consider quality of life. Such benefits are intended to be captured by health utility measures that can facilitate the calculation of quality-adjusted life-years (QALYs) [1–3]. One leading health utility measure is the EQ-5D, a generic measure of health [4, 5].

The EQ-5D-3L (“3L”) was the first iteration of the instrument. It consisted of five dimensions of health presented in the same order—mobility, self-care, usual activities, pain/discomfort, and anxiety/depression, with three severity levels per dimension describing 243 unique health states [6]. The 3L health states can be described with a five-digit numerical string, where each digit corresponds to a dimension level and ranges from 11111 (no problems on any dimension) to 33333 (extreme problems/confined to bed for all dimensions). The 3L is frequently employed in studies of population health, clinician trials, and economic evaluations; evidence of its validity is well established for many applications [7, 8].

The 3L descriptive system has been criticized for lack of sensitivity and ability to discriminate small differences in health, particularly among respondents with milder problems [8]. In response, a five-level version EQ-5D-5L (“5L”) was developed that maintained the same five dimensions as the 3L but increased the number of levels to five, thereby describing 3125 unique health states (i.e., 11111–55555) [9]. Responses to the 3L and 5L health state classifiers can be converted to an index-based utility score (value) using preference-based scoring systems derived from the general population. These value sets are typically anchored by 0 for death and 1 for full health; some health states can be valued as worse-than-dead (WTD) with negative values [7].

In comparing the properties of the descriptive systems, the 5L demonstrated improved discriminatory power and decreased ceiling effects while convergent validity and known-group validity were similar between the two descriptive systems [8]. A 2018 systematic review that compared measurement properties of the 3L and 5L descriptive systems and/or value sets [10] found support for both the 3L and 5L across patient groups and geographic locations, and the 5L demonstrated marginally improved measurement properties. More recent longitudinal and cross-sectional evidence reported consistent findings [7, 11, 12].

Because the 3L and 5L descriptive systems and their associated value sets are different, the values each system/value set produces for the purpose of cost effectiveness analysis are likely to differ. In the United States (US), three scoring approaches are of primary interest: the 3L value set by Shaw et al., the 5L–3L (5L > 3L) crosswalk value set by Van Hout et al., and the 2019 5L value set by Pickard et al. [13–15]. The 3L value set was developed first, and before the development of county-specific 5L value sets, a linking function between descriptive systems was developed to assign index values to 5L health states based on a country’s 3L value set, referred to as “5L > 3L crosswalk” or “crosswalk” thereafter in this manuscript [13]. Previous work by Law et al. did compare US 3L and 5L utility indices. However, these value sets were not the final US 3L or 5L value sets and minimized variation from other sources [16]. Since then, the final US 5L value set was published in 2019 [14]. With the availability of official value sets for the United States based on the 3L [15] and 5L [14], a comparison between published US value sets can now be completed.

The objectives of this study were to: (10 compare the normative and empirical properties of the available US EQ-5D value sets (3L, 5L, 5L > 3L crosswalk) and (2) evaluate the responsiveness of the three value sets across the complete health spectrum by use of a simulation-based method applied to cross-sectional data.

## Methods

### EQ-5D value sets

The US 3L valuation largely replicated the methods used in the UK Measurement and Valuation of Health study [15]. The 5L > 3L crosswalk served as an interim method to map 5L responses to 3L value sets prior to the availability of 5L value sets and used data from an international parallel fielding study that recruited respondents from Denmark, England, Italy, the Netherlands, Poland, and Scotland [8, 13]. The US 5L valuation employed the internationally standardized experimental design, protocol, and official platform for valuing the EQ-5D-5L [14].

### Analysis

The value sets were compared theoretically using value set characteristics and empirically using datasets in which both the 3L and 5L were administered to all respondents. All analyses were conducted in SAS 9.4 (Cary, NC) or R studio 1.3.1056 (Boston, MA).

### Theoretical value set characteristics comparison

Value sets were compared in terms of range of scale, number, and percent of health states WTD (utility < 0), mean single dimension-level utility transition, and utility difference between 11111 and the next best health state. Mean single dimension-level utility transitions were estimated by averaging all possible single-level deteriorations and improvements for a single health state described by the instrument. Such analyses were previously described by Law et al. [16]. The single dimension-level utility transition means across the range of utility values in each value set were visualized using a scatterplot to assess measurement properties. Scales with interval measurement properties can distinguish order; further, differences between adjacent values are equidistant and meaningful [17]. Approximately equal transition values across the spectrum of level sum score values would be consistent with interval measurement properties. A smoothed kernel density plot for the index values was also generated for each value set. The plots were compared in terms of distribution shape and the presence of multiple local-maximum values.

### Empirical value set characteristic comparison

#### Data sources (US 5L valuation and parallel fielding dataset)

Data that included self-completion of both the 3L and 5L from respondents that range in health were needed to facilitate comparisons of the available US value sets. The dataset from the 2017 US 5L valuation study and the 3L/5L multi-country parallel fielding study were therefore chosen for the analyses. The US 5L valuation study dataset included over 1000 respondents who were quota-sampled in terms of age, gender, race, and ethnicity to be representative of the US general population in 2017 [14]. (Table 1) The parallel fielding dataset was comprised of patients with various disease states such as diabetes and chronic obstructive pulmonary disease (Table 1) [8]. Therefore, only respondents who completed the 3L, 5L, and EQ VAS were included in the analyses.

**Table 1:** Dataset characteristics

Characteristic	Dataset	
	US valuation data	Parallel fielding data
Sample size*	1133	3790
Population	General population	Healthy and disease populations
VAS, mean (SD)	80.4 (15.6)	64.1 (22.6)
5L utility, mean (SD)	0.839 (0.209)	0.645 (0.349)
3L utility, mean (SD)	0.872 (0.156)	0.727 (0.233)
Crosswalk utility, mean (SD)	0.854 (0.140)	0.728 (0.219)
5L (11111), n (%)	354 (31.2%)	601 (15.9%)
3L (11111), n (%)	527 (46.5%)	763 (20.1%)

*SD* standard deviation, *VAS* visual analogue scale, 5L, EQ-5D-5L; 3L, EQ-5D-3L; 11111, best health state (no problems on each of the five dimensions: mobility, self-care, usual activities, pain/discomfort, anxiety/depression). For 3L, 1 represents no problems, 2 some problems, and 3 extreme problems/confined to bed. For 5L, 1 represents no problems, 2 slight problems, 3 moderate problems, 4 severe problems, and 5 extreme problems/unable to

*With complete 3L and 5L self-reported health descriptions available

#### Discriminative ability

Discriminative ability of value sets was assessed in terms of statistical efficiency using the ratio of F-statistics estimated from the analysis of variance (ANOVA) [18–20]. ANOVA models were calculated for each dataset and value set over groups of participants with differing health, i.e., general health (US valuation data only) and strata defined by responses to EQ VAS. EQ VAS was chosen as an anchoring value as it was external to the descriptive systems and was divided into ten total strata by 10 s, i.e., 0–10, 11–20…91–100. A ratio greater than 1.0 indicated the value set in the numerator had greater relative efficiency than the value set in the denominator; for all comparisons, the US 5L value set was the numerator. Data was bootstrapped with replacement to generate 1000 samples with the same sample size as the dataset to estimate 95% confidence intervals for F-statistic ratios. The ratio of respondents who reported each general health level was maintained within each sample. In the parallel fielding dataset, analyses by EQ VAS strata were also conducted within each patient group.

#### Novel simulation method for empirical responsiveness comparison

A new method was developed to understand the responsiveness of each value set across the entire spectrum of health using simulated data. The three compared value sets were applied to respondent EQ-5D health states. Then 1000 samples of 1000 respondents each were simulated using random draws (bootstrapping) with the probability of any respondent being selected varying as a function of a triangular distribution, with the top point varying over the range of possible EQ VAS values (0–100) for each dataset. Using this method, the overall severity could be varied predictably while maintaining a realistic variation between responses and minimum (0) and maximum (100) could be maintained [21]. The resulting index values were plotted for visual comparisons to determine whether measurement properties differed overall and by segments of the EQ VAS. Additional details regarding the VAS-weighted simulation are in Additional file 1: Appendix A.

## Results

### Theoretical value set characteristics

Of the three available US value sets for the EQ-5D, the 5L value set had the largest range of scale of 1.573 (vs. 1.109 for 3L and crosswalk). The 5L health state also assigned the largest percent of health states with index values less than 0, i.e., worse-than-dead. Only 1.2% and 4.1% of the health states in the crosswalk and 3L value sets were WTD compared to 19.8% of the 5L value set health states. (Table 2) The 5L value set also had the smallest utility difference between 11111 and the health state with the next highest utility value. This utility difference was 0.057, 0.112, and 0.140 for the 5L, crosswalk, and 3L value sets, respectively. The mean single-level transition across all health states was largest for the 3L value set with a mean (SD) of 0.111 (0.029). The crosswalk had the smallest mean single-level transition of 0.061 (0.017), whereas this value for the 5L was 0.078 (0.014).

**Table 2:** Theoretical value set characteristics

Characteristic	US 5L	US 3L	Crosswalk
Number of health states	3125	243	3125
Range of scale	1.573	1.109	1.109
Value for 11111	1	1	1
Number of health states WTD (%)	620 (19.8%)	10 (4.1%)	39 (1.2%)
Utility difference between 11111 and next best health state	0.057	0.140	0.112
Mean single level transition across all health states, mean (SD)	0.078 (0.014)	0.111 (0.029)	0.061 (0.017)

*SD* standard deviation, *WTD* worse than death, 5L, EQ-5D-5L; 3L, EQ-5D-3L, 11111, best health state (no problems on each of the five dimensions: mobility, self-care, usual activities, pain/discomfort, anxiety/depression). For 3L, 1 represents no problems, 2 some problems, and 3 extreme problems/confined to bed. For 5L, 1 represents no problems, 2 slight problems, 3 moderate problems, 4 severe problems, and 5 extreme problems/unable to

**Fig. 1:**
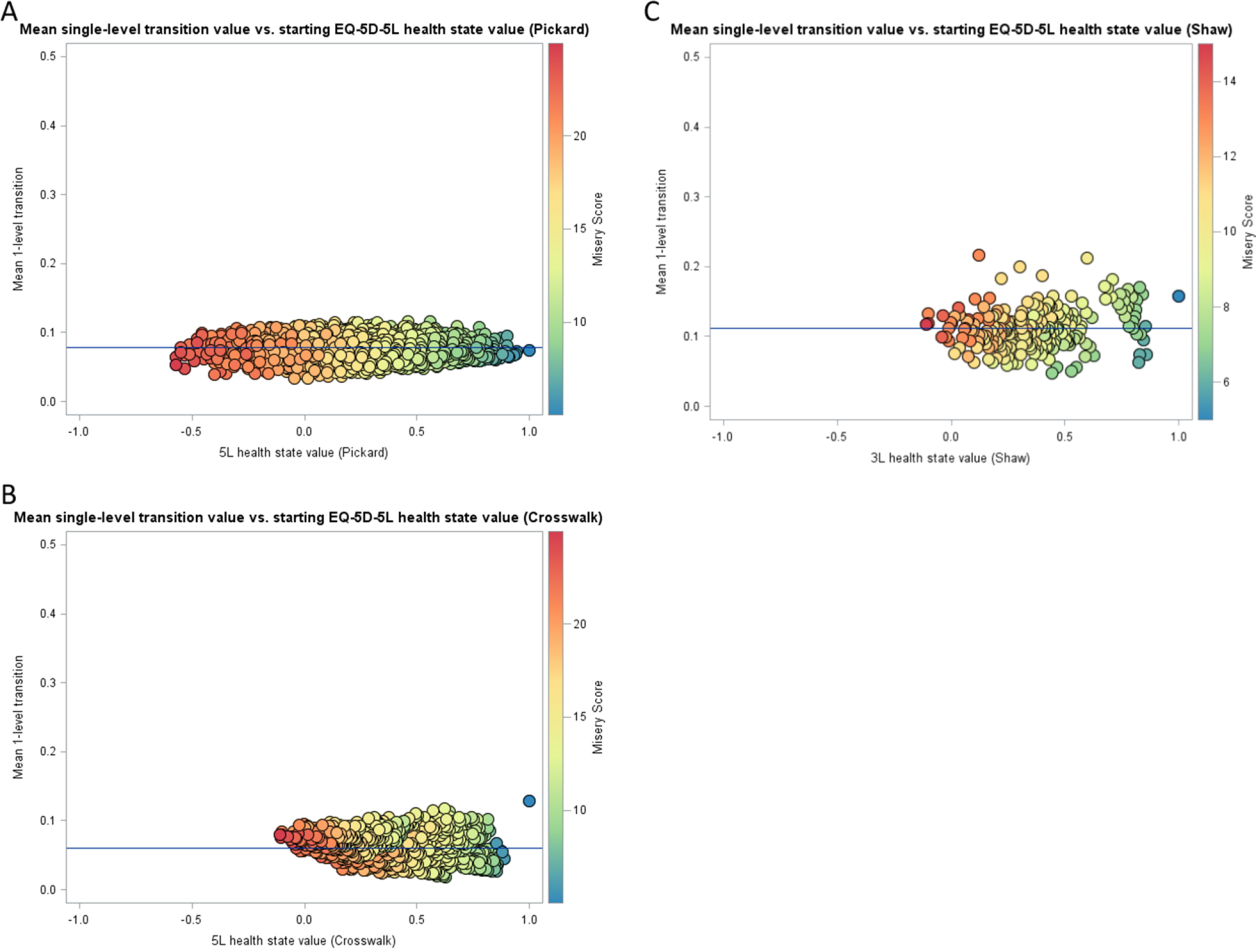
Mean single level transitions by utility of starting health state. Each panel depicts the scatterplot of mean single-level transitions for a US EQ-5D value set. The horizontal line in each graph is the mean single value transition across all single-level transitions for the plotted value set

All mean single-level transitions from each health state described by the value sets were plotted using scatter plots as a function of the starting EQ-5D index in Fig. 1. From these scatterplots, the 5L value set demonstrated improved interval measurement properties as the mean single-level transitions are closest to the mean and consistent throughout the range of health state severity as measured by level sum score. The 3L and crosswalk value sets each have a clear outlier for the mildest health state (11111) caused by the relatively large distance between 11111 and the next best health state for both value sets. The distance is 0.140 for the 3L and 0.112 for the crosswalk (Table 2).

**Fig. 2:**
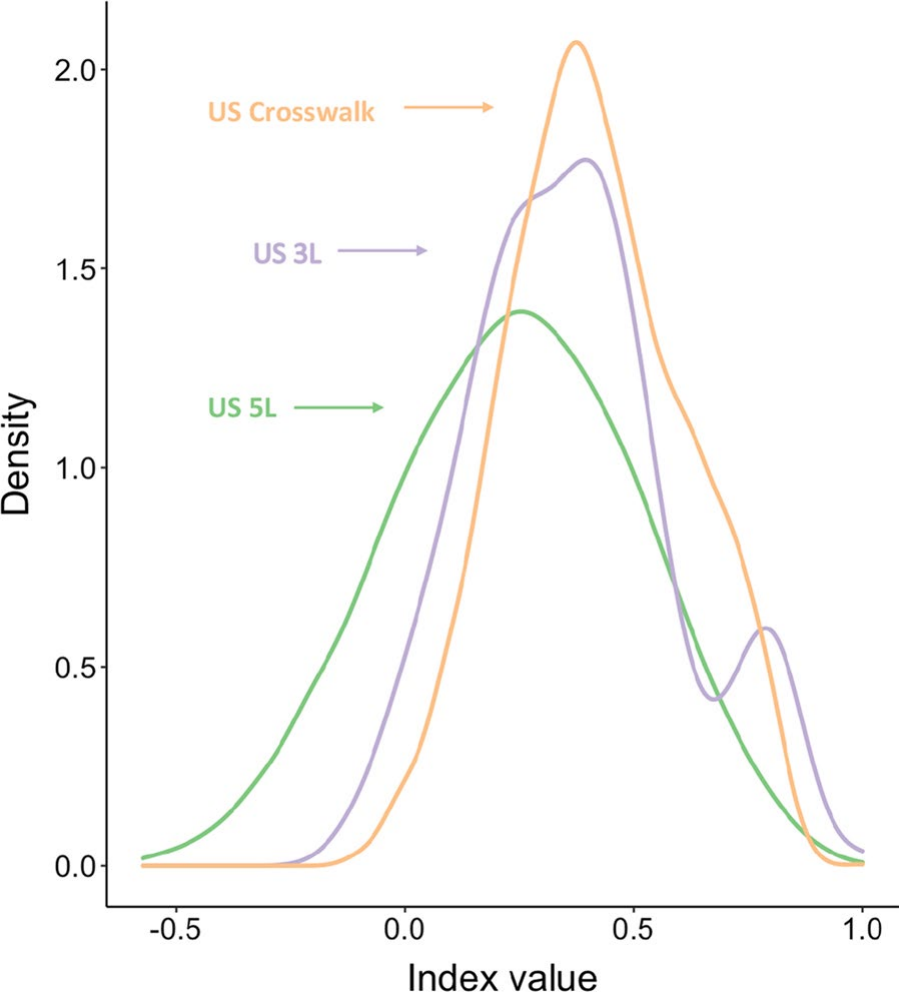
Kernel density plots. 5L—EQ-5D-5L value set (243 health states), 3L—EQ-5D-3L value set (3125 health states), crosswalk: matched 5L–3L value set (243 health states)

Furthermore, the potential for interval measurement properties was demonstrated by the smoothed kernel density plots (Fig. 2). The 5L value set distribution was closest to a normal distribution with a single maximum point, whereas the 3L value set had multiple local maxima. The crosswalk value set only had a single maximum, but the distribution was skewed.

### Empirical value set comparison

#### Discriminative ability—respondent characteristics

**Fig. 3:**
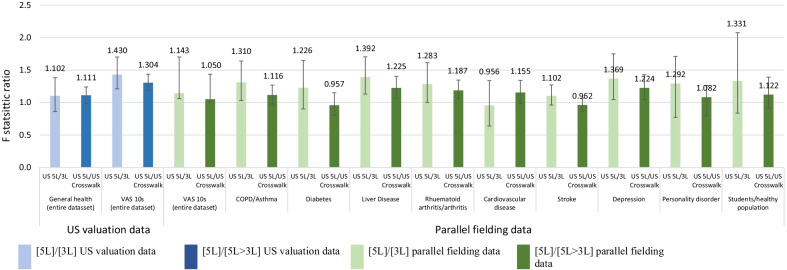
F statistic ratios by EQ VAS and self-reported health state in parallel fielding data

In terms of statistical efficiency, in the US valuation data, the 5L value set tended to be more discriminative than the crosswalk (F-statistic ratio: 1.111 95% CI 0.989–1.240) and 3L (F-statistic ratio: 1.102 95% CI 0.861–1.383) across levels of general health (Fig. 3). Furthermore, across categorical groupings of EQ VAS, the 5L was the most discriminative (F-statistic ratios 1.050–1.430) in both the US valuation and the parallel fielding datasets (Fig. 3).

Within disease states, the 5L value set was also consistently more discriminative than the 3L and crosswalk value sets for varying EQ VAS with few exceptions (Fig. 3). The crosswalk value set was more discriminating than the 5L value set in diabetes, rheumatoid arthritis/arthritis, and stroke, and F-statistic ratios were 0.981, 0.935, and 0.962, respectively. Other F-statistic ratios ranged from 1.077 to 1.513, indicating greater relative efficiency of the 5L value set over the crosswalk and 3L value sets.

#### Responsiveness—simulated utility values by EQ VAS

**Fig. 4:**
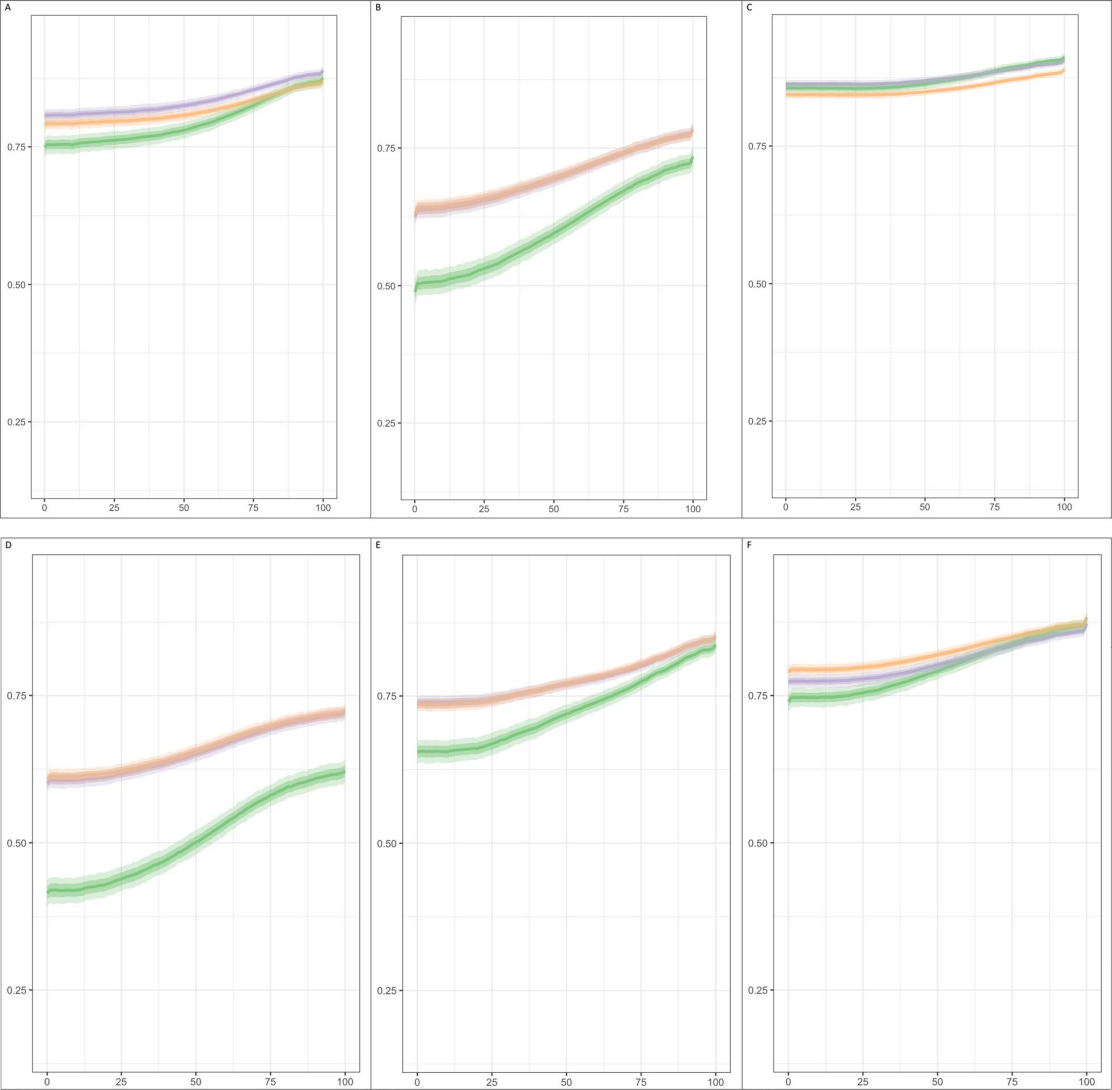

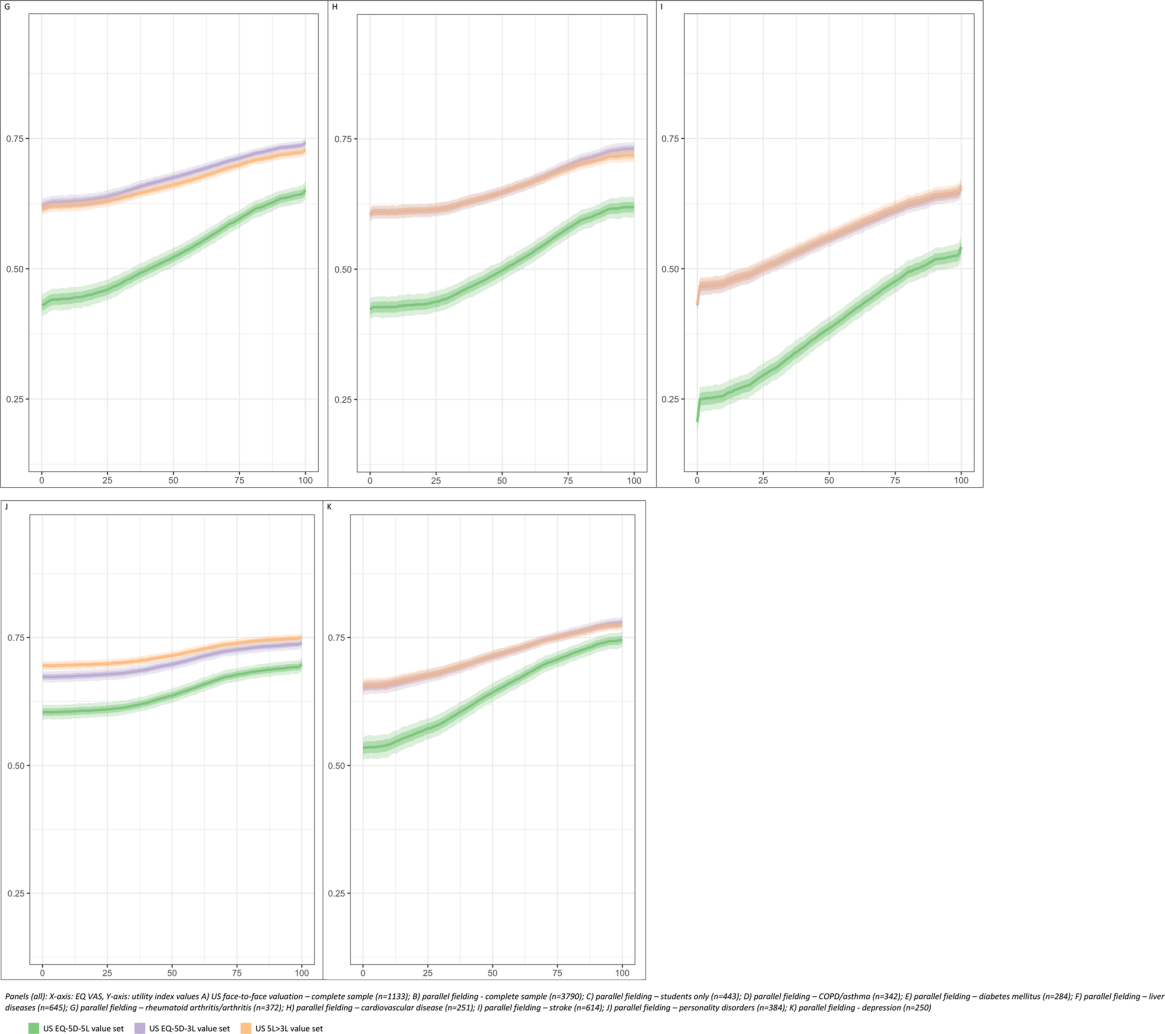
Ribbon plots for simulated mean and 95% confidence interval utility index values by value set by visual analogue value

In the US valuation dataset of general population respondents, the simulated utility values for each of the three compared value sets were similar across the range of EQ VAS values (0–100). The mean 5L utility value varied from 0.749 (95% CI 0.732–0.764) to 0.876 (95% CI 0.866–0.885) compared to the crosswalk values of 0.790 (95% CI 0.780–0.800) through 0.871 (95% CI 0.864–0.878) and 3L values of 0.806 (95% CI 0.795–0.815) to 0.889 (95% CI 0.882–0.897) (Additional file 2: Appendix B). These simulated index values were plotted as ribbon plots in Fig. 4. For each value set pictured in Fig. 4, the dark solid line represents the average simulated index value at a given EQ VAS. The medium shading and light shading represented the interquartile range and the 95% confidence interval of the simulated index values, respectively. In the US valuation dataset, the simulated utility values were similar across the entire spectrum of EQ VAS values for all three value sets (Fig. 4a).

Larger utility differences were noted between value sets in the parallel field dataset. The mean 5L utility value ranged from 0.489 (95% CI 0.465–0.512) through 0.734 (95% CI 0.716–0.750) compared to the crosswalk values range of 0.630 (95% CI 0.616–0.645) to 0.783 (95% CI 0.771–0.793), and US 3L values ranged from 0.625 (95% CL 0.609–0.641) to 0.784 (95% CI 0.772–0.795). (Additional file 2: Appendix B) In the student group of the parallel fielding dataset, the three value sets produced closer utility values across the EQ VAS spectrum (Fig. 4c).

For all health conditions in the parallel fielding dataset, the 5L value set produced lower utility values than the 3L and crosswalk value sets for all EQ VAS values (Figs. 3d–k). For health conditions such as rheumatoid arthritis/arthritis, cardiovascular disease, and depression, the 5L value set may be more discriminative across different levels of health and/or responsive to changes in health. In most health conditions, the 5L index values changed more rapidly with differences with EQ VAS, i.e., steeper slope, between VAS values of 25 and 75. This trend is less evident in stroke and personality disorders (Additional file 2: Appendix B, Fig. 4).

This study represents a key addition to the literature in comparing the available US EQ-5D value sets and also introduces a novel simulation method for empirical responsiveness comparison across the entire spectrum of health using cross-sectional data. These results demonstrated that the US 5L value set had more desirable theoretical and empirical measurement properties than the US 3L and crosswalk value sets. The improved interval measurement properties of the 5L were supported by the scatterplots of the mean individual-level transitions and kernel density plots of index values (Fig. 2). These figures highlight key benefits of the 5L value set—consistent, predictable transitions between adjacent health states across the entire scale. The crosswalk value set had the smallest mean single-level transition of the three value sets, but this observation can be attributed to many health states (3125) over a shorter range of scale (1.109). Related to both the value set range of scale and the increased levels of severity in its descriptive system, the US 5L value set was found to be generally more discriminative than the 3L and crosswalk value sets in both datasets.

The 5L was also the most responsive of the three value sets; within the simulation analyses, the responsiveness of value sets was most distinct between EQ VAS values of 25 and 75, with the steeper slope of the 5L value set demonstrating greater responsiveness. The slopes of the compared value sets were similar between low (0–25) and high (75–100) EQ VAS values, and responsiveness distinctions were less conclusive in patients with poor and good health, respectively. However, if the discriminative ability is used as a proxy measure for responsiveness, the 5L was found to be more discriminative in the students’ group of parallel fielding data and the US valuation respondents in terms of F-statistic ratios. These can be considered as two healthy groups similar to patients with EQ VAS greater than 75. Therefore, a key shortcoming of the 3L (i.e., decreased sensitivity to change) in healthier patients may be addressed by 5L and the corresponding value set [9]. An evidence gap remains in understanding the measurement properties of US value sets in patients with very poor health. This could not be pursued in the current analyses as a few severely ill (i.e., had EQ VAS values < 50) patients were included in the empirical datasets.

This study builds upon the Law et al. study through the application of the official US value sets using a novel method to compare instrument/value set performance [16]. The increased discriminatory ability of the 5L identified in this study is generally consistent with findings in other countries and studies, including a recent empirical head-to-head comparison of value sets for multiple countries [7, 16, 22]. However, previous evidence comparing responsiveness to change between value sets is mixed—some studies reported 5L had improved responsiveness while others found no or even reduced responsiveness [12, 23–25]. These discrimination and responsiveness findings may be disease state and/or geographically dependent [26, 27]. Further evaluations of value set responsiveness in specific disease states using longitudinal data may be limited by the lack of such data availability. The novel, simulation-based method outlined in this study can be applied to cross-sectional data to investigate the responsiveness of the value sets across the entire health spectrum (e.g., EQ VAS 0–100). This method enables broader insight than previous studies by showing the relative performance of measures/value sets across a broad range of levels of health. In this way, our results and future application of this method to other datasets can help to inform choice of measure and value sets prior to clinical trial initiation. The method may also be extended to comparisons of other instruments if health anchors external to the instruments’ descriptive systems is included in addition to the other instruments.

Based on these findings, general consequences of the choice of descriptive system and/or value set for health measurement and cost-effectiveness may be identified. The 5L instrument and its US value set can better distinguish patients with different levels of health. Additionally, changes between 5L index values over time may be greater than changes measured using the 3L and crosswalk value sets when anchored on EQ VAS changes. The 5L value set index values are more sensitive to changes or differences in health. These larger utility differences for improvements in health may also result in a lower incremental cost-effectiveness ratio if survival benefits are similar between comparators.

This study was limited by the few available data sets with 3L and 5L responses provided by the same respondent. These analyses were also not conducted using trial data or longitudinal data; however, evaluation using such datasets would constrain results to only the observed changes whereas these analyses provide evidence on how changes in underlying health may be reflected in index values and potential implications for QALYs across the entire spectrum of observed health. The responsiveness analyses were only conducted using EQ VAS as an anchor; additional evaluations are necessary to confirm these findings using other measures of health. The analyses were all conducted using the EQ VAS administered following the 5L descriptive system; an “order effect” may be present where the EQ VAS value was influenced by the descriptive system administered immediately prior [11]. However, the 3L EQ VAS was not available in both datasets used, so sensitivity analyses could not be conducted.

## Conclusion

In conclusion, the US 5L value set had longer range of scale, increased precision in health status measurement, and tended to be more discriminative than summary scores based upon the 3L value set and crosswalk. The greater sensitivity to health changes of the 5L value set over the full range of health would potentially produce lower incremental-cost effectiveness ratios than scores based on the 3L. The simulation method can facilitate comparisons of sensitivity of different value sets and/or utility measures in patient groups and populations when only cross-sectional data is available.

## Supplementary Information

**Additional file 1.** Triangular weighting function explanation.

**Additional file 2.** Additional descriptive statistics of simulation results.

## Data Availability

Datasets used in this study may be available upon request to the corresponding author.
