# Supplementary material for: EQ-5D-5L measurement properties are superior to EQ-5D-3L across the continuum of health using US value sets
Source: Health Qual Life Outcomes. 2022 Sep 9;20:134. doi: 10.1186/s12955-022-02031-8 (PMC9463847; doi:10.1186/s12955-022-02031-8)

**Appendix A – Triangular weighting function explanation**

When simulating data by sampling a continuous variable, a triangular weighting function *w* may be applied across that variable’s range. The weighting function can be described according to the below formula*:*

$$w\left( x,v,lb,ub \right)=min(\frac{x-lb}{v-lb},\frac{x-ub}{v-ub})$$

In this formula, *lb* and *ub* are the lower and upper bounds of the triangle, which can be set at the minimum and maximum values of the continuous variable. *x* is a value to be weighted in the continuous variable, and *v* is the value corresponding to the top of the weighting distribution.

In the weighting function, v will receive a relative weight of 1, indicating that persons/items in the sample with a value of *v* for the variable have the highest chance of being selected. The triangle is repeated for each value of *v* across the *lb* and *ub* continuum. For example, if the *lb* is 0 and *ub* is 100, there are 101 triangle distributions used for 0-100, inclusive, and each distribution has a different value­ for *v*  (i.e., 0, 1, 2,…98, 99, 100). This allows for the simulation of datasets which covers the entire spectrum of the continuous variable.

R code:

triangular_weights <- function(x, v, lb = 0, ub = 100)

mapply(function(a, b) min(a, b),

a = (x-lb)/(v-lb),

b = (x-ub)/(v-ub))

Below is a plot of weights for *x* ranging from 0 to 100 by steps of 1, with a *v* of 75, *lb* of 0, and *ub* of 100:

plot(triangular_weights(0:100, 75)


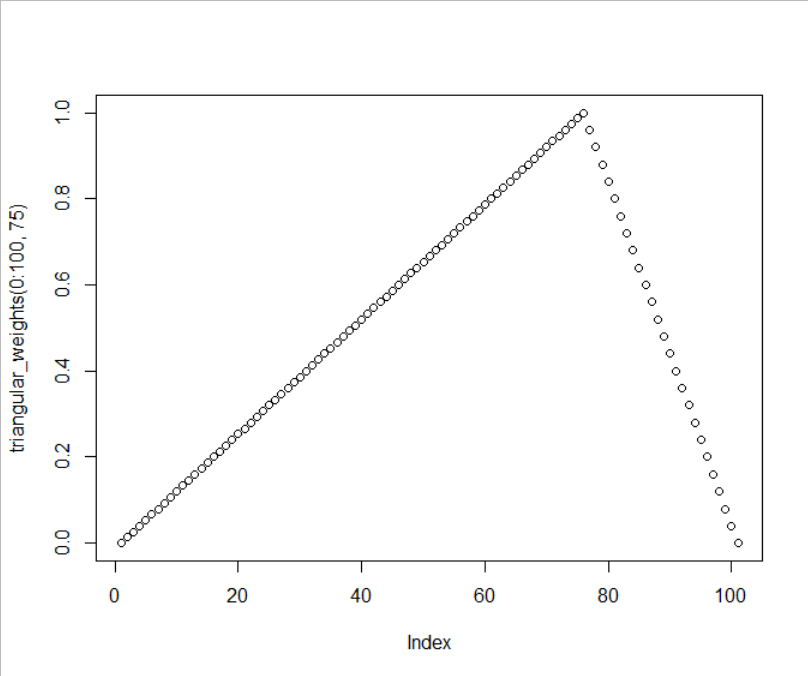

Supplement: Supplementary file 1 — Additional file 1. Triangular weighting function explanation. [file 12955_2022_2031_MOESM1_ESM.docx]
